# Artificial intelligence in ophthalmology

**DOI:** 10.22336/rjo.2023.37

**Published:** 2023

**Authors:** Stella Ioana Popescu (Patoni), Alexandra Andreea Mihaela Muşat, Cristina Patoni, Marius-Nicolae Popescu, Mihnea Munteanu, Ioana Bianca Costache, Ruxandra Angela Pîrvulescu, Ovidiu Mușat

**Affiliations:** * Department of Ophthalmology, “Dr. Carol Davila” Central Military Emergency University Hospital, Bucharest, Romania; ** Department of Ophthalmology, “Victor Babeş” University of Medicine and Pharmacy, Timişoara, Romania; *** “Carol Davila” University of Medicine and Pharmacy, Bucharest, Romania; **** Department of Gastroenterology, “Dr. Carol Davila” Central Military Emergency University Hospital, Bucharest, Romania; ***** Physical and Rehabilitation Medicine, Elias Emergency University Hospital, Bucharest, Romania; ****** Department of Ophthalmology, Bucharest Emergency University Hospital, Bucharest, Romania

**Keywords:** artificial intelligence, macular holes, age-related macular degeneration, diabetic retinopathy, glaucoma, etinopathy of prematurity

## Abstract

One of the fields of medicine in which artificial intelligence techniques have made progress is ophthalmology. Artificial intelligence (A.I.) applications for preventing vision loss in eye illnesses have developed quickly. Artificial intelligence uses computer programs to execute various activities while mimicking human thought. Machine learning techniques are frequently utilized in the field of ophthalmology. Ophthalmology holds great promise for advancing artificial intelligence, thanks to various digital methods like optical coherence tomography (OCT) and visual field testing. Artificial intelligence has been used in ophthalmology to treat eye conditions impairing vision, including macular holes (M.H.), age-related macular degeneration (AMD), diabetic retinopathy, glaucoma, and cataracts. The more common occurrence of these diseases has led to artificial intelligence development.

It is important to get annual screenings to detect eye diseases such as glaucoma, diabetic retinopathy, and age-related macular degeneration. These conditions can cause decreased visual acuity, and it is necessary to identify any changes or progression in the disease to receive appropriate treatment.

Numerous studies have been conducted based on artificial intelligence using different algorithms to improve and simplify current medical practice and for early detection of eye diseases to prevent vision loss.

**Abbreviations: **AI = artificial intelligence, AMD = age-related macular degeneration, ANN = artificial neural networks, AAO = American Academy of Ophthalmology, CNN = convolutional neural network, DL = deep learning, DVP = deep vascular plexus, FDA = Food and Drug Administration, GCL = ganglion cell layer, IDP = Iowa Detection Program, ML = Machine learning techniques, MH = macular holes, MTANN = massive training of the artificial neural network, NLP = natural language processing methods, OCT = optical coherence tomography, RBS = Radial Basis Function, RNFL = nerve fiber layer, ROP = Retinopathy of Prematurity, SAP = standard automated perimetry, SVP = Superficial vascular plexus, U.S. = United States, VEGF = vascular endothelial growth factor

## Artificial intelligence

Artificial intelligence involves using computers’ ability to perform various tasks. These artificial intelligence devices can use their language to develop new strategies, learn, and reason. By synthesizing this information, these devices perform tasks with minimal human effort. In medicine, medical activity is much more precise and efficient by using artificial intelligence, which has the role of imitating human thinking [**1**,**2**].

There are three types of artificial intelligence:

1. General artificial intelligence - this type of intelligence, theoretically, can perform the tasks that human intelligence can perform. 

2. Narrow general intelligence - this type of intelligence performs tasks such as human intelligence, but in a narrow range.

3. Artificial superintelligence - this intelligence is superior to human thinking in all areas and activities [**1**,**3**,**4**].

In many medical specialties, artificial intelligence is constantly growing. It uses different devices, applications, and algorithms to prevent, diagnose and treat conditions. Two major types of devices are used:

1. Natural language processing methods (NLP) that have the role of extracting information from unstructured data, such as medicated journals, and medical notes.

2. Machine learning techniques (ML) that analyze structured data, such as genetic data and images, represent a secondary screening and diagnosis technique. Machine learning techniques allow computers to make predictions by repeatedly learning existing materials [**2**,**5**,**6**,**14**].

According to the research, the applications employed can help identify people with vision loss that can be avoided by visiting an ophthalmologist. Artificial intelligence applications for preventing vision loss in eye illnesses have developed quickly [**1**,**5**,**7**]. 

Artificial intelligence techniques utilized in ophthalmology fall into two categories:

1. Because deep learning uses numerous layers to produce the outcome, predictions can be made more accurately. In addition to accomplishing the classification objective, this technique has the advantage of extracting the features appropriate for a specific situation [**1**,**8**].

In deep learning, two algorithms are commonly used: the massive training artificial neural networks (MTANN) and the convolutional neural network (CNN). Both processes try to identify and categorize photos. In ophthalmology, CNNs are used to categorize and segment pictures from the retina [**8**-**10**].

2. Self-learning algorithm creates results using a single neural network layer. It mimics the nervous system’s neuronal architecture by developing artificial neural networks (ANNs). Because ANNs have limitations, data must be entered to identify the model. Machine learning’s function is to create an algorithm using data entered into the computer. The method is then applied to enhance predictions [**1**,**8**].

There are two types of machine learning: supervised learning and non-secondary learning. Ophthalmology is an attractive field of artificial intelligence development through various digital techniques, such as visual field testing, optical coherence tomography (OCT), and images from the back of the eye. Applications of artificial intelligence in ophthalmology have focused on eye diseases that lead to vision loss, such as age-related macular degeneration, diabetic retinopathy, glaucoma, and cataracts. The increased prevalence of these diseases in the United States has led to an artificial intelligence approach [**8**,**11**-**13**].

OCT pictures monitor and diagnose retinal illnesses such as macular holes, diabetic macular edema, and age-related macular degeneration. Deep learning methods are used in this study to automatically read OCT pictures. These formulas are continually being improved [**8**,**11**,**12**,**15**].

## The role of Artificial Intelligence in managing Age-Related Macular Degeneration (AMD)

As we age, we may experience a chronic and irreversible eye condition called age-related macular degeneration. This condition is one of the primary causes of central vision loss. It is characterized by changes in the retinal, drusen, exudates, hemorrhage, and neovascularization at the choroid level [**2**,**16**-**18**].

The appearance of drusen is a central feature of age-related macular degeneration. Drusen is classified into two types, hard and soft, present inside or outside the pigment retinal epithelium and can be seen on OCT images. Through machine and deep learning, artificial intelligence can diagnose age-related macular degeneration as early as possible by automatically detecting the drusen of fluids. Applications based on artificial intelligence have demonstrated increased sensitivity and specificity, leading to improved diagnosis treatment by early diagnosis [**19**-**22**]. Some algorithms can predict visual acuity from age-related macular degeneration and the need for anti-VEGF treatment. Others can identify the condition through OCT images or color images of the fundus [**23**]. 

A study conducted by Brulina and her collaborators states that an automated assessment using deep learning of age-related macular degeneration is consistent with manual assessment. These automated algorithms are vital in managing and monitoring this condition in addressing screening costs and new treatments [**19**,**24**].

OCT is one of the most critical investigations in the detection and prognosis of age-related macular degeneration, especially the wet form requiring anti-VEGF treatment by intravitreal injection [**25**].

Antony and his collaborators used a CNN algorithm, called VGG16, to diagnose this macular condition in OCT scans. This CNN algorithm has two parts; one is called an encoder that encodes all the essential features of the image in the last feature map. This map is flattened into a list of numbers called a vector passed to FFNN. This algorithm can specify the likelihood of a patient developing age-related macular degeneration [**26**,**27**].

Schlegl and his collaborators developed a deep learning-based algorithm for identifying and quantifying macular edema in OCT images caused by age-related macular degeneration. This method has an average accuracy of 0.94, optimal accuracy, and superior performance in identifying intraretinal fluid in AMD [**19**,**28**]. 

Peng and his collaborators developed a deep learning-based application, called DeepSeeNet, which assesses the risk of progression and severity in late AMD by automatically classifying it from color photos of the fundus. DeepSeeNet has superior precision and accuracy to retinal specialists in the early detection of pigment abnormalities and drusen characteristics of age-related macular degeneration [**19**,**29**].

These studies based on artificial intelligence using different algorithms have demonstrated superior specificity and sensitivity. Shortly, these applications will be commonly used in diagnosing and treating age-related macular degeneration [**19**].

## Artificial intelligence in glaucoma

Glaucoma is a medical condition in which the pressure inside the eye increases because of a blockage in the drainage of the fluid, known as aqueous humor, which leads to damage to the optic nerve. Worldwide, it is the second leading cause of vision loss. Currently, OCT is used to evaluate glaucoma, which automatically measures the thickness of the retinal nerve fiber layer (RNFL) and ganglion cell layer (GCL) by using the automatic artificial intelligence segmentation technique [**19**,**26**,**30**-**32**]. According to recent studies, deep learning techniques can effectively detect glaucoma and its changes with greater accuracy and speed [**33**-**37**].

Currently, two forms of injuries were cited by the American Academy of Ophthalmology (AAO), structural and functional lesions. Functional lesions are visual field defects, while structural lesions include optic disc abnormalities or damage to the RNFL. Deep learning methods have a role in glaucoma screening by increasing the identification of biomarkers and risk factors [**38**,**39**]. 

Sigueoka and his collaborators conducted a study on 124 patients, using standard automated perimetry (SAP) and OCT as deep evaluation methods to discern between individuals with glaucoma and healthy ones. R.F. and FFNN were used as ML algorithms to calculate model standard deviation, and mean deviation from SAP and to extract characteristics by measuring RNFL at different points in the image and for calculating. The Radial Basis Function (RBS) network uses special activation functions and is the most powerful model. This ML technique is essential for diagnosing and treating glaucoma, especially with no specialists [**26**,**40**]. 

Another recent study by Mariottoni, in 2020, demonstrated that deep learning algorithms can detect relevant B-scan characteristics to predict RNFL thickness without the segmentation of retinal layers. The disadvantage of this algorithm is represented by the presence of a high rate of artifacts compared to OCT [**33**,**41**-**43**]. Thompson’s study is much more accurate, using a CNN model to circumvent the limitation produced by the technique described by Mariottoni. CNN’s algorithm uses peripapillary B scans to predict the likelihood of glaucoma [**33**,**44**].

Jammal and his team developed an M2M DL deep learning algorithm to compare its effectiveness with that performed manually by glaucoma specialists in identifying cup-to-disc ratio and thickness changes in RNFL. This algorithm analyzed 490 fundus photographs for cup-to-disc ratio estimates and the likelihood of glaucomatous optic neuropathy. The results were compared with those achieved with the SAP algorithm’s significantly superior performance. The deep learning method by M2M DL is needed in glaucoma screening to identify glaucomatous optic neuropathy and reduce the retinal nerve fiber layer [**38**,**45**].

Evaluating the optic nerve head’s (ONH) integrity is essential in detecting glaucoma. Due to the anatomical variation of the optic nerve head, it is challenging to detect optic disc lesions clinically and by screening. Recently, artificial intelligence has made progress in identifying glaucomatous optic disc slugs. The input layer represented by an optic nerve image is assigned a truth label, such as “glaucoma” or “glaucoma-free”. The hidden output layer becomes the input for the next hidden layer. During deep learning, the algorithm identifies properties in an image and assigns a classification label to the output layer [**46**-**49**].

In 2022, Aktar and his collaborators developed the first technique for identifying glaucoma, considering both risk factors and functional and structural data. This algorithm trains three DL models by calculating cup surface area and demonstrates that it is an essential parameter in the future in detecting glaucoma [**50**,**51**].

The artificial intelligence approach can simplify and improve the identification of glaucoma patients, but it has certain limitations. It requires a large amount of data to train DL models, which is quite difficult and time-consuming. More studies are needed to demonstrate clinical utility [**50**,**51**]. 

## Artificial Intelligence in Diabetic Retinopathy

Diabetic retinopathy, an ischemic eye condition, is the most common complication in patients with type 1 diabetes. This disease is the leading cause of vision loss in patients with long-standing diabetes. It is characterized by retinal neurodegeneration, hemorrhages, and microaneurysms in the early stages and does not show obvious symptoms. In advanced stages, microvascular abnormalities and cotton wool spots appear. Annual screening for diabetic retinopathy is essential, regardless of the stage of the disease, to prevent vision loss [**52**-**57**].

The U.S. Food and Drug Administration (FDA) approved in 2018 an AI-based algorithm, IDx, developed to identify diabetic retinopathy [**53**,**58**,**59**]. It is designed to work for non-mydriatic fundus with the Topcon NW 400 camera. This system uses one image centered on the optical disc and one on the macula for each eye for analysis and needs all four images to display a result [**60**,**61**].

Previous versions of IDX-DR were studied as part of the Iowa Detection Program (IDP). These versions used separate algorithms to detect exudates, hemorrhages, neovascularization, cotton wool stains, and irregular strokes and to quantify images. A study conducted on the Caucasian, North African population aimed to analyze 3640 images of the fundus of patients based both on the IDP algorithm and by ophthalmologists. In 334 cases, image quality needed to be improved. IDP specificity was 70%, and IDP sensitivity was 86.7% [**60**,**62**-**64**].

The IDX-DR system enhanced the IDP by adding DL-based features. To see if this system offered an advantage, it was checked with a Messidor 2 dataset. Specificity was improved to 80% and thus reduced the number of false positives, while sensitivity remained unchanged [**60**,**65**]. 

In Portugal, RetmarkerDR software was developed for the local screening of diabetic retinopathy. The main feature of this system is its ability to compare previously performed images with current images, establishing the progression of the disease. RetmarkerDR exhibits the so-called “microaneurysm rotation rate”, which implies that the system can identify the rate of disappearance of old microaneurysms and the rate of microaneurysm formation [**60**,**66**].

Other AI-based systems, such as EyeArt, and Bosch DR, for diabetic retinopathy screening, require several additional studies to reach a result [**60**].

Early identification and treatment of diabetic retinopathy is a priority in preventing vision loss in patients with diabetes. Many AI-based screening systems with superior sensitivity and specificity have been developed [**60**].

## The Role of Artificial Intelligence in Detecting Retinopathy of Prematurity (ROP)

Retinopathy of prematurity is the leading cause of vision loss in children worldwide. Screening for retinopathy of prematurity based on the application of artificial intelligence can detect early signs of severe prematurity retinopathy and prevent blindness with appropriate treatment. Brown developed the i-ROP DL system for the early diagnosis of this condition. It may produce a severity score to demonstrate disease progression, regression, and response to treatment [**53**,**67**-**70**].

## Artificial Intelligence in Macular Holes

The macula is the area of the retina responsible for clear, detailed central vision.

A macular hole occurs as a tear in the macula.

The amount to which a hole will impair someone’s eyesight depends on size and location.

To diagnose a macular hole, a comprehensive dilated eye exam is necessary as it enables the evaluation of the condition of the macula. An OCT scanner examines the retina and the macula in detail by scanning the back of the eye.

By analyzing photographs of the eye and developing a customized surgical plan based on the size of the macular hole, AI can help with surgical planning.

The size of macular holes measured on OCT can not only predict recovery, but OCT-A can also be helpful by submitting to the analysis of Superficial vascular plexus (SVP) and deep vascular plexus (DVP), whose morphological changes can predict visual acuity [**71**].

## Conclusions

Artificial intelligence is the process of employing a computer’s processing power to carry out numerous activities. Artificial intelligence, which has the potential to improve medical performance significantly, is used in diagnostic accuracy.

Most AI-based systems are used in developed countries, and some require further study. High costs and lack of doctors and equipment in undeveloped countries and rural areas make eye disease screening challenging. In the future, screening and early detection of ophthalmic diseases through artificial intelligence techniques will become routine in medical practice.

**Conflict of Interest statement**

The authors declare no conflict of interest.

**Acknowledgments**

None.

**Sources of Funding**

No support was needed.

**Disclosures**

None.
